# From Fermented Sugar to Incretin Therapeutics: Reuniting Metabolism and Addiction

**DOI:** 10.1016/j.bpsgos.2026.100821

**Published:** 2026-09-22

**Authors:** Mark S. Gold, Daniel M. Blumenthal

**Affiliations:** a Department of Psychiatry, Washington University School of Medicine in St. Louis, St. Louis, Missouri; b Cardiology Division, Mayo Clinic Florida, Jacksonville, Florida; c Cardiovascular Associates of America, Celebration, Florida

In a recent study published in *Biological Psychiatry: Global Open Science*, Reitz *et al.* (1) provide a timely and ambitious review of glucagon-like peptide-1 receptor (GLP-1R) agonists and glucose-dependent insulinotropic polypeptide receptor (GIPR)/GLP-1R dual agonists at the intersection of alcohol use disorder (AUD), obesity, and cardiometabolic disease. Their chief contribution is not simply to identify another possible medication for AUD but also to argue that alcohol-related and metabolic disease converge along shared gut-brain-liver pathways. Selected patients may be treated using the same neuroendocrine system. This would be a welcome shift from treating drinking, AUD, weight, diabetes, and liver injury as separate end points and moving toward treating the whole person in whom they exist together.

This convergence has a longer scientific history than the current incretin era suggests. Incretins are metabolic hormones, GLP-1 and GIP, released by the gut after one eats, signaling satiety. One of these authors’ (MSG) own paths went from addiction neuroscience to obesity research. Working alongside colleagues at Princeton and Yale, they began with the recognition that drugs and food are not identical reinforcers but rather recruit overlapping systems that govern reward, motivation, satiety, and loss of control (2). Early work showed that intermittent sugar access could produce bingeing, withdrawal-like behavior, craving, and repeated dopamine release in the nucleus accumbens—evidence that hyperpalatable, energy-dense foods can engage addiction-like circuitry even though food itself is not a drug of abuse (3). We and others have placed these observations amid a broader model linking homeostatic hormones, mesolimbic reward, and executive control (4).

At the University of Florida, Gatorade offered an instructive precedent. Its success depended on physiology—replacing water, carbohydrates, and electrolytes—as well as on palatability, which drove repeated consumption. Ingestive behavior is never determined by calories or pharmacology alone. Taste, learning, and reinforcement determine whether a hyperpalatable substance, sweet or intoxicating, is used again (5). The journey from Gatorade to GLP-1 medicines connects 2 eras of translational physiology: designing an ingestible rehydration product and using gut-derived signals to modify appetite, reward, and behavior.

The relevance to alcohol is important and is more than metaphorical. Alcohol, the product of fermented fruit, grain, or sugar cane, is pharmacologically distinct from sugar. But alcohol carries energy, alters glucose and lipid metabolism, and is consumed within learned sensory and social contexts. Fermentation converts a carbohydrate substrate into a psychoactive molecule, layering intoxication, tolerance, and organ toxicity onto an already potent ingestive cue—one reason the boundary between food reward and drug reward is biologically porous, even when the clinical disorders remain distinct. Therefore, Reitz *et al.* (1) are right to focus on patients affected by both alcohol and metabolic risk. Treatments that reduce drinking and improve weight, glycemic control, and hepatic fat could simultaneously interrupt several mutually reinforcing disease processes.

### What Has Changed With GLP-1R Agonists?

The early food addiction literature evaluated whether medications developed for substance addiction could reduce pathological overeating and whether obesity treatments could reduce drug seeking. In a commentary published in *Biological Psychiatry* in 2013, Avena and Gold (6) argued that animal models supported precisely this two-way strategy. GLP-1R agonists now provide the most compelling test of that hypothesis. GLP-1 is not simply a satiety signal; peripheral and central GLP-1 systems influence gastric emptying, insulin secretion, vagal afferent signaling, stress responses, and mesolimbic reward, offering several nonexclusive routes to reduced drinking. Critically, the therapeutic signal should produce clinically beneficial and persistent alcohol-specific outcomes.

New work from d’Ávila *et al.* (7) adds an unanticipated layer to this circuitry. In female mice, chronic semaglutide activated agouti-related peptide (AgRP) neurons rather than silencing them, and genetic removal or inhibition of these classic hunger neurons prevented the drug from sustaining its weight-lowering effect. The finding shows that GLP-1R agonists do more than suppress appetite and may recruit the brain’s adaptive response to caloric deficit to coordinate fat loss. However, there is no existing evidence indicating that the same AgRP pathway mediates reduced alcohol use. But it strengthens the hypothesis that incretin therapeutics act through dynamic, distributed gut-brain networks rather than through a simplistic satiety switch.

Human evidence has moved beyond anecdote. Low-dose weekly semaglutide reduced laboratory alcohol self-administration and craving in adults with AUD, though not every prespecified consumption measure (8), and a more recent trial in patients with AUD and comorbid obesity reported reductions in heavy-drinking days and drinking-risk level (9). These findings establish a clinically important proof of concept. The obesity signal may be especially informative: an earlier exenatide trial was negative for its primary AUD outcome but suggested a benefit in participants with obesity (1). AUD is heterogeneous, and an incretin medication may prove valuable not because it works uniformly but because it works best in a biologically enriched phenotype—obesity, insulin resistance, metabolic liver disease, or strong ingestive craving.

### Why Dual Agonism Is Promising—and Still Unproven

The move from GLP-1R to GIPR/GLP-1R dual agonism is plausible but not automatic. Tirzepatide, a dual agonist, produces greater weight loss and glycemic improvement than GLP-1R agonists such as semaglutide. However, greater metabolic potency may not yield greater anti-addiction efficacy; the independent contribution of GIPR signaling to alcohol reinforcement is unknown. As Reitz *et al.* (1) acknowledge, no completed randomized trial has established that tirzepatide treats AUD. The supporting evidence—drug-target Mendelian randomization linking genetically proxied GLP-1R and GIPR agonism to lower binge and high-risk drinking (10), observational prescription data, and preclinical work—is hypothesis generating. However, it cannot readily rule out confounding by weight loss, treatment selection, or nausea. Still, GLP-1R agonists have crossed the threshold from mechanistic promise to preliminary randomized evidence in AUD; dual agonists have not. The review is strongest when it frames dual agonism as a testable next-generation hypothesis rather than an established treatment.

### A Translational Agenda—and a Clinical Warning

Trials should recruit the population in whom integrated benefit is most plausible—AUD plus obesity, type 2 diabetes, or metabolic and alcohol-related liver disease—comparing a dual agonist with placebo and, when feasible, a GLP-1R agonist and an active naltrexone arm. Incretin therapy should be studied as an addition to, not a replacement for, behavioral treatment and established medications such as naltrexone, acamprosate, or disulfiram. Who is to say that naltrexone plus GLP treatment is not better than either alone?

Trials that include patients with physiologic alcohol dependence, who are at risk for withdrawal, which can be life-threatening, must incorporate mechanisms for proactively preventing and treating withdrawal symptoms. Understanding the trajectory of change in alcohol consumption based on treatment dosing will be critically important for anticipating and proactively mitigating this risk.

Naltrexone is the appropriate benchmark and a warning. It reliably reduces alcohol-related harms, craving, and relapse but is markedly underused in practice; even when prescribed, adherence to daily oral dosing is often poor, and injectable formulations have not solved the problem of retention (10). Fewer than 1 in 10 patients likely to benefit from an AUD medication currently receive one—not because our treatments fail, but because physicians infrequently screen for or prescribe them, and stigma keeps patients from asking for or taking their prescriptions.

Medication efficacy and treatment impact are not the same thing. GLP-1R and dual agonists may, however, have several advantages that naltrexone never had, such as weekly dosing; initiation and follow-up care can occur in a primary care setting rather than in addiction or mental health settings, and patients may accept and continue to take this medication prescribed for diabetes, weight, or cardiometabolic health even when reducing alcohol use was not their original goal. That alternate route into AUD treatment could lower stigma and broaden reach—but it should be measured, not assumed, and it does not excuse the field from addressing why a genuinely effective medication such as naltrexone still reaches so few of the patients who need it.

Cardiovascular, metabolic, and liver outcomes—including glycemic control, hepatic steatosis, fibrosis, and cardiometabolic outcomes—deserve particular attention. Outcomes should go beyond body mass index and self-reported alcohol consumption. Five-year studies should assess drinking through timeline follow-back measures, phosphatidylethanol and other objective biomarkers, and heavy-drinking days. They should also evaluate treatment retention, medication adherence, and the durability of benefits after discontinuation.

We know that patients with AUD have higher rates of coronary artery disease, atrial fibrillation, and obstructive sleep apnea (OSA). However, we also know that GLP-1R and GLP-1/GIPR agonists treat cardiovascular risk factors—including diabetes, hyperlipidemia, blood pressure, and inflammation—by slowing and even reversing disease progression (e.g., by slowing coronary plaque progression and improving OSA)—and reduce rates of major adverse cardiovascular events. Safety and implementation must receive equal scrutiny, including the risks of pancreatitis, malnutrition, and sarcopenia; sex-specific effects; psychiatric comorbidity; and risks associated with detoxification or rapid reductions in drinking. These concerns are especially important because patients with AUD are often poorly served by systems organized to treat either addiction or metabolic disease, but rarely both.

Fifty years of translational research on food and addiction have taught us that discoveries rarely move in a straight line from molecule to treatment. Instead, they travel from bedside observation to biological mechanism—and back to the bedside to help carefully selected patients (2). The incretin story is now following that path in an unexpected direction: a treatment developed for metabolic disease is challenging fundamental assumptions about reward and addiction. Reitz *et al.* (1) have mapped the next stage of this journey not to relabel AUD as obesity or alcohol as food but to consider how fermented carbohydrates, brain reward, metabolism, and alcohol-related organ injury converge in some patients and to ensure that whatever we learn ultimately reaches that patient.
